# The Ovarian Transcriptome at the Early Stage of Testis Removal-Induced Male-To-Female Sex Change in the Protandrous Black Porgy *Acanthopagrus schlegelii*

**DOI:** 10.3389/fgene.2022.816955

**Published:** 2022-03-23

**Authors:** Peng-Wei Tseng, Guan-Chung Wu, Wei-Lun Kuo, Yung-Che Tseng, Ching-Fong Chang

**Affiliations:** ^1^ Doctoral Degree Program in Marine Biotechnology, National Taiwan Ocean University, Keelung, Taiwan; ^2^ Doctoral Degree Program in Marine Biotechnology, Academia Sinica, Taipei, Taiwan; ^3^ Department of Aquaculture, National Taiwan Ocean University, Keelung, Taiwan; ^4^ Center of Excellence for the Oceans, National Taiwan Ocean University, Keelung, Taiwan; ^5^ Marine Research Station, Institute of Cellular and Organism Biology, Academia Sinica, Taipei, Taiwan

**Keywords:** ovotestis, sex determination, sexual fate, sex differentiation, sex change, hermaphroditic fish, estrogen

## Abstract

Unlike gonochoristic fishes, sex is fixed after gonadal differentiation (primary sex determination), and sex can be altered in adults (secondary sex determination) of hermaphroditic fish species. The secondary sex determination of hermaphroditic fish has focused on the differences between testicular tissue and ovarian tissue during the sex change process. However, comprehensive studies analyzing ovarian tissue or testicular tissue independently have not been performed. Hermaphroditic black porgy shows a digonic gonad (ovarian tissue with testicular tissue separated by connective tissue). Protandrous black porgy has stable maleness during the first two reproductive cycles (<2 years old), and approximately 50% enter femaleness (natural sex change) during the third reproductive cycle. Precocious femaleness is rarely observed in the estradiol-17β (E_2_)-induced female phase (oocytes maintained at the primary oocyte stage), and a reversible female-to-male sex change is found after E_2_ is withdrawn in <2-year-old fish. However, precocious femaleness (oocytes entering the vitellogenic oocyte stage) is observed in testis-removed fish in <2-year-old fish. We used this characteristic to study secondary sex determination (femaleness) in ovarian tissue *via* transcriptomic analysis. Cell proliferation analysis showed that BrdU (5-bromo-2′-deoxyuridine)-incorporated germline cells were significantly increased in the testis-removed fish (female) compared to the control (sham) fish (male) during the nonspawning season (2 months after surgery). qPCR analysis showed that there were no differences in pituitary-releasing hormones (*lhb* and *gtha*) in pituitary and ovarian steroidogenesis-related factors (*star*, *cyp11a1*, *hsd3b1*, and *cyp19a1a*) or female-related genes (*wnt4a*, *bmp15*, *gdf9*, *figla*, and *foxl2*) in ovarian tissues between intact and testis-removed fish (2 months after surgery). Low expression of pituitary *fshb* and ovarian *cyp17a1* was found after 2 months of surgery. However, we did find small numbers of genes (289 genes) showing sexual fate dimorphic expression in both groups by transcriptomic analysis (1 month after surgery). The expression profiles of these differentially expressed genes were further examined by qPCR. Our present work identified several candidate genes in ovarian tissue that may be involved in the early period of secondary sex determination (femaleness) in black porgy. The data confirmed our previous suggestion that testicular tissue plays an important role in secondary sex determination in protandrous black porgy.

## Introduction

**FIGURE 1:**
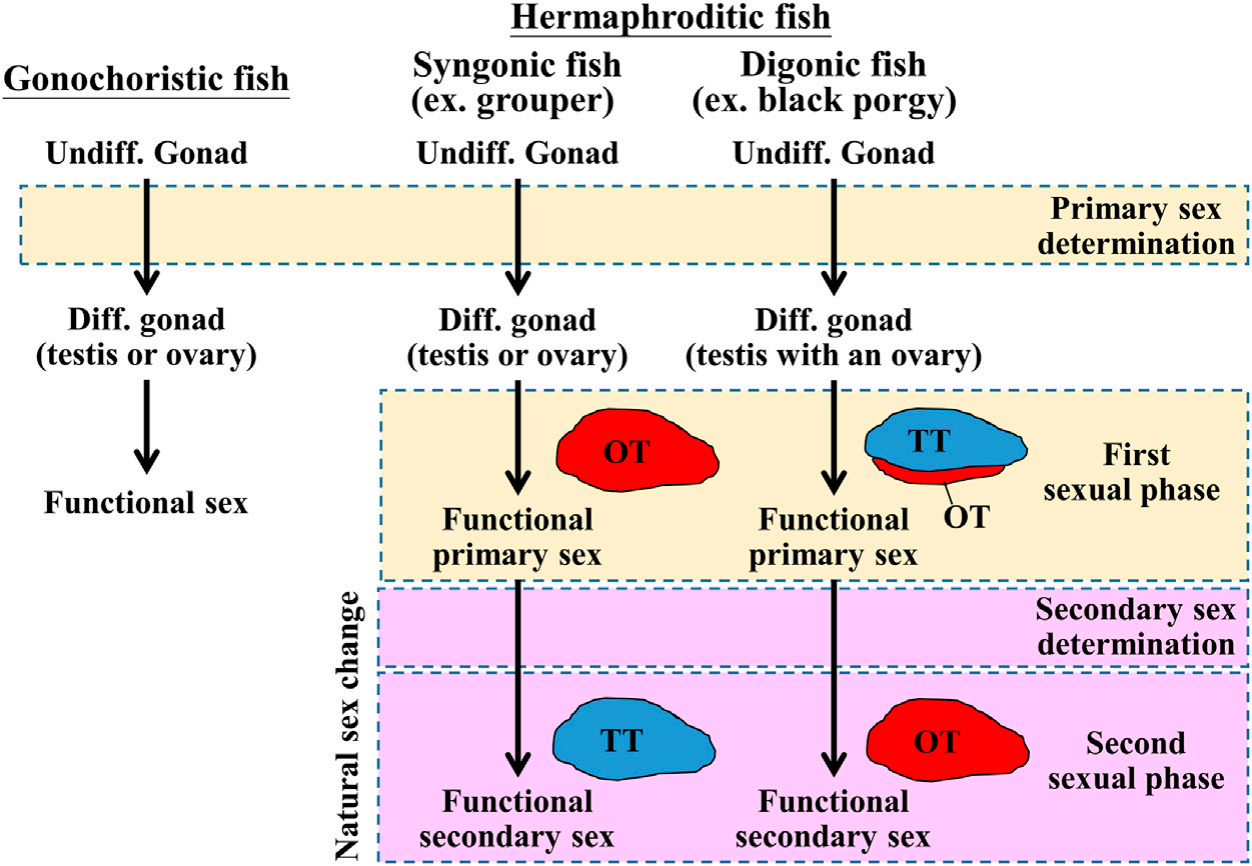
Schematic diagram of sex determination and gonadal differentiation in teleosts. Sex determination is a process to influence sexual fate. In gonochoristic fish, sex determination resulted in undifferentiated gonad to differentiated gonad (testis or ovary). Then the testis or ovary develops into a mature phase (functional sex). In syngonic fish, primary sex determination resulted in undifferentiated gonad to differentiated gonad, then the ovary develops into a mature phase (functional primary sex). In the natural sex change process, secondary sex determination caused primary sex to secondary sex (male-to-female or female-to-male). In digonic fish, such as black porgy, primary sex determination resulted in the change of undifferentiated gonad to differentiated gonad (a bisexual gonad, testis with an ovary), then the gonad develops into functional primary sex (mature testis with regressed ovary). In the natural sex change process, secondary sex determination caused primary sex to secondary sex. Undiff. gonad, undifferentiated gonad; Diff. gonad, differentiated gonad.

In gonochoristic fishes, sex is determined by the interaction of different factors, such as genetic factors and environmental factors (Bachtrog et al., 2014; Lin et al., 2021). Unlike gonochoristic fishes, which have fixed sexes after sex determination/gonadal differentiation, approximately 6% of teleost fish species exhibit functional (fertile) hermaphroditism (De Mitcheson and Liu 2008). Most hermaphroditic fishes are sequential hermaphrodites whose sex is altered while the fish reach a certain size or age. According to their first functional sex, hermaphroditic fishes are divided into protogyny (female-to-male sex change), protandry (male-to-female sex change), and serial bidirectional sex change (Munday et al., 1998; Todd et al., 2016). According to their gonadal structure, gonads are divided into syngonic gonads (no clear barrier between ovarian and testicular tissues) and digonic gonads (ovarian and testicular tissue separated by connective tissue) by histology. The differences in sex determination, gonadal differentiation, and development between gonochoristic and hermaphroditic (syngonic and digonic gonad) fishes are shown in Figure 1. Unlike gonochoristic fishes, in which sex is fixed after gonadal differentiation (primary sex determination), the sexual phase can be altered in adults (secondary sex determination and secondary sexual phase, also called sex change) of hermaphroditic fish species. In protogynous grouper (*Epinephelus* spp.) (Zhou and Gui 2010; Guo et al., 2021) and protandrous black porgy (Chang and Yueh 1990; Chang et al., 1997), secondary sex determination is determined by a certain age or body size, respectively. In protogynous bluehead wrasse (*Thalassoma bifasciatum*) (Warner and Swearer 1991), protandrous anemone fish (*Amphiprion* spp. *and Premnas biaculeatus*) (Fricke and Fricke 1977; Godwin 1994), and bidirectional sex change coral-dwelling gobies (Gobiodon spp. and Paragobiodon spp.) (Todd et al., 2016), sex change is determined by social cues.

The gonadotropin-gonadotropin receptors (*luteinizing hormone/chorionic gonadotropin receptor, lhcgr*, and *follicle-stimulating hormone receptor, fshr*) are considered to regulate reproductive function in teleosts (review in Zohar 2021). In medaka (*Oryzias latipes*) (Takahashi et al., 2016) and zebrafish (*Danio rerio*) (Chu et al., 2015), *fshb* (*follicle-stimulating hormone subunit beta*) knockout affects folliculogenesis and causes a smaller oocyte diameter than in wild-type fish. *fshr* knockout causes a small ovary and infertility in medaka (Murozumi et al., 2014) and zebrafish (Chu et al., 2015). In female zebrafish, the *lhb* (*luteinizing hormone subunit beta*) mutant is infertile, but the *lhcgr* mutant is fertile (Chu et al., 2014). Exogenous Gths causes precocious sex changes in bluehead wrasse (human chorionic gonadotropin, hCG) (Koulish and Kramer 1989), orange-spotted grouper (*Epinephelus coioides*, porcine FSH) (Huang et al., 2019), tiger grouper (*Epinephelus fuscoguttatus*, rFsh) (Palma et al., 2019), honeycomb grouper (*Epinephelus merra*, bovine FSH) (Kobayashi et al., 2010), and ricefield eel (*Monopterus albus*, mammalian LH) (Tang et al., 1974). In contrast, administration of exogenous Gths in black porgy (hCG) (Du et al., 2005) and honeycomb grouper (bovine LH) (Kobayashi et al., 2010) could not induce precocious sex changes. Taken together, these data demonstrate species-specific Gnrhs-Gths signaling in teleosts. Other brain factors can also regulate sex changes in fish. A study in protogynous bluehead wrasse showed that the sex change could be controlled by social signals, as removal of the male fish caused the largest female fish sex change (Warner and Swearer 1991). Furthermore, transcriptomic studies suggest that dynamic cortisol production and signaling (hypothalamic-pituitary-interrenal axis) could be a key factor in sex in bluehead wrasse (Liu et al., 2015; Todd et al., 2019). Cortisol can stimulate *hsd11b2* (*11β-hydroxysteroid dehydrogenase*) expression and then produce 11-ketotestosterone (11-KT) and cortisone (Fernandino et al., 2013). Estradiol-17*β* (E_2_) is the key sex steroid controlling female fate. Aromatase (encoded by *cyp19a1*) is a key enzyme involved in E_2_ synthesis that is important to ovarian differentiation, development, and growth in fish. Inhibition of E_2_ synthesis by an aromatase inhibitor (AI) in early development can induce testicular development in salmon (Piferrer et al., 1994), rainbow trout (Guiguen et al., 1999), Japanese flounder (Kitano et al., 2000), tilapia (*Oreochromis niloticus*) (Afonso et al., 2001), and black porgy (Lee et al., 2002). In addition to *cyp19a1a*, genes such as *star* (*steroid acute regulatory protein*), *cyp11a* (*cytochrome P450 side-chain cleavage enzyme*), *cyp17a* (*17α-hydroxylase/C-17−C-20 lyase*), and *hsd3b* (*3β-hydroxysteroid dehydrogenase/Δ5, Δ4-isomerase*) are related to sex steroid hormone biosynthesis and play crucial roles during gonadal development (Tenugu et al., 2021).

Protandrous black porgy (*Acanthopagrus schlegelii*) is an important marine aquaculture fish species. Fish with sex differentiation occurring at the age of 3–4 months have a stable primary male phase during the first two reproductive cycles (<2 years old), and approximately 50% of fish alter their sexual phase from male to female (secondary sexual phase, also called sex change) during the third reproductive cycle (Chang and Yueh 1990; Chang et al., 1997). The ovarian and testicular tissues of the gonad are well separated from the connective tissue in black porgy (Lee et al., 2008, Lee et al., 2011). Black porgy has been recognized as a unique fish model to study the endocrine and genetic aspects of the mechanisms of sex differentiation and sex change (Chang et al., 1995a; Chang et al., 1995b; Chang and Lin 1998; Lee et al., 2000; Lee et al., 2001; Wu et al., 2005; Wu et al., 2010b). Plasma E_2_ levels were significantly increased during the process of natural sex change in 2–3-year-old black porgy (Chang et al., 1995b). Sex steroids (E_2_, testosterone), aromatase, and aromatase inhibitor were applied to manipulate sex differentiation and sex changes in black porgy (Chang et al., 1995a; Chang et al., 1995b; Lau et al., 1997; Chang and Lin 1998; Lee et al., 2002; He et al., 2003a; He et al., 2003b; Du et al., 2003; Lee et al., 2004; Wu et al., 2008b).

Precocious femaleness is rarely observed in the E_2_-induced female phase (oocytes remained at the primary oocyte stage), and a reversible female-to-male sex change is found after E_2_ is withdrawn in <2-year-old fish (Chang and Lin 1998; Lee et al., 2004; Wu et al., 2008a). However, precocious femaleness (oocytes entering the vitellogenic oocyte stage and/or maturation) is observed in testis-removed fish in <2-year-old fish (Wu et al., 2008a; Wu et al., 2016). Higher plasma luteinizing hormone (Lh) levels were observed in males than in females during natural sex changes in 2–3-year-old black porgy (Du et al., 2005). The gene expression levels of *fshr* and *lhcgr* were significantly higher in testicular tissue than in ovarian tissue (Wu et al., 2016). Furthermore, increasing gene expression levels of *fshr* and *lhcgr* were found in testicular tissue from the nonspawning to prespawning seasons of the second reproductive cycle (Wu et al., 2016). However, the expression profiles of *fshr* and *lhcgr* in the ovarian tissue did not change in the primary male phase or the testis removal-induced secondary female phase from the nonspawning to prespawning season of the second reproductive cycle (Wu et al., 2016). Moreover, testicular *dmrt1* (*doublesex and mab-3 related transcription factor 1*) transcripts were significantly increased by *in vivo* LHRH analog and *in vitro* hCG (Wu et al., 2012). In contrast, *in vivo* hCG could not change the *cyp19a1a* expression profile in ovarian tissue (Wu et al., 2016). These results show that the male phase, but not the female phase, could be induced by Gnrh-Gths signaling in black porgy. Thus, the secondary sex determination of black porgy may be determined through the regulation of the hypothalamus-pituitary-testis axis in black porgy (Wu and Chang 2018; Wu et al., 2021).

As mentioned above, the fate of the secondary female phase was controlled by the testicular tissue in protandrous black porgy, but the molecular mechanism of the ova-testis interaction was not clear. To understand the molecular mechanism of the ova-testis interaction on the secondary sex determination/early period of the female phase, we compared the differences of ovarian tissue between control (sham) maleness and testis removal-induced femaleness through RNA-seq and transcriptomic analysis. We characterized the morphological differences in both groups by histological study. We further examined the gonadal proliferative activity of ovotestis in control (sham) fish and ovaries in testis-removed fish by BrdU (5-bromo-2′-deoxyuridine)-incorporating assay (immunohistochemical staining). We also analyzed the gene expression profiles of ovarian differentiation/development-related genes by qPCR, including pituitary-releasing hormones (*fshb*, *lhb*, and *gtha*) in pituitary and ovarian steroidogenesis-related factors (*star*, *cyp11a1*, *hsd3b1*, *cyp17a1*, and *cyp19a1a*) and female-related genes (*wnt4a*, *bmp15*, *gdf9*, *figla*, and *foxl2*). We carried out transcriptomic analysis of ovarian tissue in control (sham) maleness and testis removal-induced femaleness. Our data identified that the first characteristic of femaleness was a higher number of proliferating germline cells in the testis-removed femaleness compared to the control (sham) maleness status 2 months after surgery. Our results identified several candidate genes in ovarian tissue that may be involved in the early period of secondary sex determination (femaleness) in black porgy.

## Materials and methods

### Animals

The experimental fish was acclimated to a seawater pond environment (2.5-ton FRP tank) at the National Taiwan Ocean University aquaculture station. All fish were cultured with a natural lighting system and water temperature. All procedures and investigations were approved by the National Taiwan Ocean University Institutional Animal Care and Use Committee and were performed in accordance with standard guidance principles.

### Experimental design

Black porgies have stable maleness during the first two spawning seasons. However, precocious femaleness was observed in the testis-removed fish during the second spawning season in our previous study (Wu et al., 2008a). To examine the secondary sex determination/female phase of ovarian tissue between ovarian development and the presence or absence of a testis, we surgically removed the testicular part of the digonic gonad and then chip-labeled the fish to determine their sexual phase (maleness or femaleness) during the second reproductive season (<2 years old). We incised an abdominal cavity between the pectoral fin and urogenital pore (approximately 20% of body length) and held the ovotestis and then clearly removed the testicular part of ovotestis. The testicular tissue was separated from the ovotestis with a small portion of ovarian tissue. After removing the testicular part of ovotestis, we stitched the incision of abdominal cavity and applied antibiotics (oxolinic acid) to the incision. The fish were incubated in the seawater with antibiotics (30 ppm oxolinic acid) for 3 days (renewed the antibiotics daily). In >1-year-old fish, the testis was removed by surgery during the nonspawning season (status 7, dominant ovarian tissue with regressed testicular tissue) in the testis-removed fish (*n* = 12; body length = 23.0 ± 2.2 cm). In the control (sham) fish, surgery was performed, but the testicular part of the digonic gonad was not removed (*n* = 16; body length = 22.3 ± 2.6 cm). The gonads of both groups were collected 1 month (for transcriptome) and 2 months (histology and qPCR) after surgery for analyses. Furthermore, the proliferative activity of gonadal cells was determined in a BrdU incorporation assay. Two months after surgery (both groups were at status 7), the fish (*n* = 12; body length = 22.5 ± 2.1 cm) were treated with BrdU (30 mg/kg of body weight) by intraperitoneal injection on Days 1 and 4, and the gonads were collected on Day 7. The gonadal status of control (sham) fish and testis-removed fish were observed to enter maleness (status 5) and femaleness (status 10), respectively. The gonadal status was divided into seven different statuses in black porgy, as shown in Supplementary Figure S1.

### Gonadal histology

Histology was performed according to previous methods (Wu and Chang 2009). The gonads from all fish were fixed with 4% paraformaldehyde in PBS (phosphate-buffered saline) at 4°C for 16 h. The fixed gonads were dehydrated in methanol and stored at −20°C. Dehydrated gonads were transferred from methanol to ethanol and then embedded in paraffin. The gonadal sections (5 µm thickness) were rehydrated and stained with hematoxylin-eosin (H&E).

### Immunohistochemical staining

Immunohistochemical (IHC) staining was performed according to previously described methods (Wu and Chang 2009). For immunohistochemical staining, the rehydrated sections (5-µm thickness) were treated with HistoVT One (06380-05, Nacalai Tesque, Kyoto, Japan) to expose the antigens of the target protein. The sections were incubated with 3% H_2_O_2_ in phosphate buffer with saline (PBS). To expose with BrdU, sections were incubated with 2 N HCl for 15 min. To detect BrdU-expressing cells and Sertoli cells expressing Dmrt1, anti-BrdU (catalog: MAB4072, Merck Millipore Inc., Darmstadt, Germany; diluted 1:1,000 with 1.5% nonfat milk powder), and anti-Dmrt1 (diluted 1:1,000 with 1.5% nonfat milk powder; Wu et al., 2012) were used. The specificity of the anti-Dmrt1 antibody was confirmed in a previous study (Wu et al., 2012). For secondary antibody reactions, biotinylated goat anti-mouse antibody (BA-9200, Vector Laboratories Inc., Burlingame, CA, USA; diluted 1:1,000 with 1.5% nonfat milk powder) and goat anti-rabbit antibody (BA-1000, Vector Laboratories Inc.; diluted 1:1,000 with 1.5% nonfat milk powder) were used for anti-BrdU and anti-Dmrt1 antibodies, respectively. Immunoreactivity was amplified with an ABC kit (PK-6100, avidin-biotin; Vector Laboratories, Inc.) and colorized by 3,3′-diaminobenzidine (DAB; D-5637-1G, Sigma, St. Louis, MO, USA). Slides were counterstained with hematoxylin.

### Cell proliferation assay

To evaluate cell proliferative activity, BrdU was used to trace cell division. BrdU-incorporated cells were determined by IHC with anti-BrdU antibody. Tissue sections were used to count the BrdU-incorporated oogonia and the total number of oogonia. The oogonia were identified based on morphological characteristics and relative cell size. The size (diameter) of the oogonia ranged 8–12 µm. The percentage of oogonia proliferation was calculated as (BrdU incorporated oogonia/total oogonia) × 100%.

### Total RNA extraction

Samples were homogenized in TRIzol reagent (Invitrogen, Waltham, MA, USA), and then total RNA extractions were performed following the manufacturer’s protocol. Total RNA was then quantified using a NanoDrop™ 1,000 spectrometer (Thermo Fisher Scientific, Waltham, Massachusetts, USA).

### Quantitative real-time PCR analysis

Four micrograms of total RNA from each sample was used to synthesize first-strand cDNA with Superscript III reverse transcriptase (Invitrogen) and oligo (dT)12–18 primers (Promega, Madison, WI, USA) following the manufacturer’s protocol. The first-strand cDNA was used for quantitative real-time PCR (qPCR) analysis. Specific primers for *gapdh* (*glyceraldehyde-3-phosphate dehydrogenase*; GenBank accession no. DQ399798), *fshb* (*follicle-stimulating hormone subunit beta*; GenBank accession no. HQ202975), *lhb* (*luteinizing hormone subunit beta*; GenBank accession no. HQ202976), *gtha* (*glycoprotein hormones alpha chain*; GenBank accession no. EF605275), *star* (*steroid acute regulatory protein*; GenBank accession no. AY870248), *cyp11a1* (*cytochrome P450 side-chain cleavage enzyme*; GenBank accession no. AY870246), *hsd3b1* (*3β-hydroxysteroid dehydrogenase/Δ^5^,Δ^4^-isomerase*; GenBank accession no. AY870247), *cyp17a1* (*17α-hydroxylase/C-17-C-20 lyase*; GenBank accession no. AY870249), *cyp19a1a* (P450arom gonad form; GenBank accession no. AY273211), *wnt4a* (*wingless-type MMTV integration site family, member 4a*; GenBank accession no. DQ268648), *bmp15* (*bone morphogenetic protein 15*; GenBank accession no. KY427737), *gdf9* (*growth differentiation factor 9*; GenBank accession no. KY427738), *figla* (*factor in the germline alpha*; GenBank accession no. EU496494), *foxl2* (*forkhead box l2*; GenBank accession no. EU496493), *fshr* (*follicle-stimulating hormone receptor*; GenBank accession no. AY598753), and *lhcgr* (*luteinizing hormone/chorionic gonadotropin receptor*; GenBank accession no. AY596169) are listed in Supplementary Table S1. Gene quantifications of standards, samples, and controls were conducted by qPCR (CFX Connect™ Real-Time PCR Detection System; Bio–Rad Laboratories, Hercules, CA, USA) with SYBR Green Master Mix (Bio–Rad Laboratories). The PCR specificity was confirmed by a single melting curve of unknown samples and standards. The relative expression pattern was normalized to *gapdh*, and the highest value of each gene in the control group was defined as one.

### Illumina sequencing

Ovarian tissue from control fish (n = 3, maleness) and testis-removed fish (n = 3, femaleness) was used for RNA-seq. cDNA synthesis, cDNA library construction, and Illumina sequencing were performed by Genomics, Inc. (New Taipei City, Taiwan). RNA-seq libraries were constructed using the Illumina TruSeq Stranded mRNA Library Prep Kit (Illumina, San Diego, CA, USA). Paired-end (150-bp) transcriptome sequencing was performed on the Illumina NovaSeq 6000 platform.

### *De novo* transcriptome assembly

Six raw data points were uploaded to the Galaxy platform, and we used the tools at usegalaxy.org to analyze the data (Jalili et al., 2020). Trimmomatic was used to remove adapters and low-quality reads (ILLUMINACLIP = TruSeq3-PE.fa:2:30:10:8, SLIDINGWINDOW = 4:5, LEADING = 5, TRAILING = 5, MINLEN = 25). The clean reads of six samples were pooled together for *de novo* assembly with Trinity v2.9.1 (Grabherr et al., 2011). Quality assessment of the *de novo* assembled transcriptome was performed by computing the ExN50 statistic and BUSCO (Simão et al., 2015). The RNA-seq data were submitted to the NCBI SRA database and released (BioProject ID PRJNA767932).

### Differential expression analysis

The transcript abundance of clean reads from each sample was estimated by Salmon (Patro et al., 2017). Statistically significant differentially expressed genes (DEGs) were identified by DESeq2 software (Love et al., 2014). The genes were considered to be DEGs with “adjusted p-value <0.05” and “|log_2_Fold Change| >1”.

### Transcriptome Annotation

TransDecoder was used to identify putative open reading frames (ORFs) from the assembled transcripts. Predicted peptides were annotated to the public database eggNOG v5.0 by eggNOG-mapper v2 (Huerta-Cepas et al., 2019; Cantalapiedra et al., 2021). The tool can be used to find the most similar orthologous groups and annotated to Gene Ontology (GO) and Kyoto Encyclopedia of Genes and Genomes (KEGG).

### Data analysis

The data are expressed as the mean ± SD. Differences between the control group and testis-removed group were assessed by Student’s *t* test, with p < 0.05 indicating a significant difference.

## Results

### Comparison of gonadal status in control maleness and testis removal-induced femaleness during the second reproductive cycle

**FIGURE 2:**
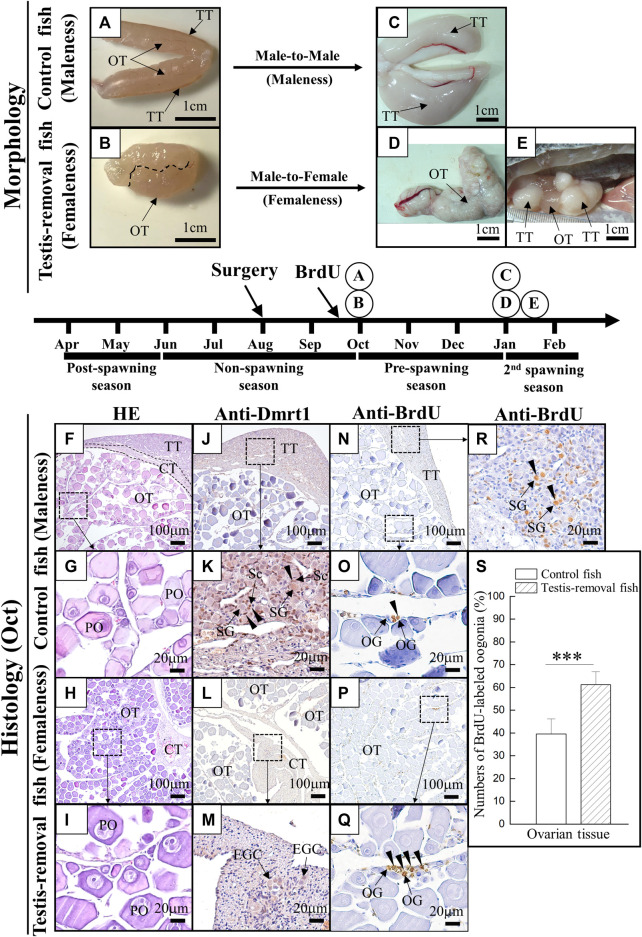
The morphology and histology analysis during testis removal-induced sex change in black porgy. In 1-year-old fish, testis excision was performed during the non-spawning season (Aug). We collected gonad during the pre-spawning season (2 months after surgery, Oct) for cell proliferation/histology **(F–S)** and second spawning season (Jan) for morphological analysis **(A–E)**. For cell proliferation assay, BrdU (30 mg/kg BW) was injected on Day 7 and Day 3 before gonadal tissue was collected (2 months after surgery, Oct). Sertoli cell-expressing Dmrt1 was used to identify the testis-removed fish has a remnant testicular tissue or not. The gonad of control fish **(A)** and testis-removed fish **(B)** were collected at 2 months after surgery. The dash line **(B)** represents the branch of the pair gonad. The gonad of control fish **(C)** and testis-removed fish **(D)** were collected during the second spawning season. **(E)** The testis-removed fish has a remnant testicular tissue during the second spawning season. **(F–I)** H&E staining of the gonad that was collected at 2 months after surgery from the control fish and testis-removed fish. **(G)** and **(I)** are the high-magnification images of **(F)** and **(H)**, respectively. **(J–M)** Gonadal IHC staining of Dmrt1 in the control fish and testis-removed fish (2 months after surgery). **(K)** and **(M)** are the high-magnification images of **(J)** and **(L)**, respectively. **(N–R)** Gonadal IHC staining of BrdU in the control fish and testis-removed fish (2 months after surgery). **(O,R)** and **(Q)** are the high-magnification images of **(N)** and **(P)**, respectively. **(S)** The results indicate that the oogonia have significantly increased proliferating activity in the testis-removed fish compared with the control fish. TT, testicular tissue; OT, ovarian tissue; CT, connected tissue; OG, oogonia; PO, primary oocyte, EGC, early germ cell; SG, spermatogonia; Sc, Sertoli cell. Asterisks indicate significant differences by Student’s *t*-test (***, p < 0.001).

Fish (>1 year old) were used in this study. In control (sham) fish, bisexual gonads had the dominant ovarian tissue with regressed testicular tissue during the nonspawning season (Figure 2A). The ovaries developed well in the testis-removed fish after 2 months of testis removal (Figure 2B). During the spawning season, mature testicular tissue with regressed ovarian tissue was found in the control (sham) fish (Figure 2C). In contrast, mature ovarian tissue was found in the testis-removed fish (Figure 2D). However, remnant testicular tissue of the bisexual gonad resulted in mature testicular tissue with immature ovarian tissue during the spawning season in testis-removed fish (Figure 2E). These results showed that the precocious femaleness (male-to-female sex change) was induced during the second spawning season through complete testis removal.

H&E staining results showed that the ovaries developed well with the primary oocytes in the control fish (Figures 2F,G) and testis-removed fish (Figures 2H,I) 2 months after surgery. No difference in oocyte size was found in the control fish (70.14 ± 9.75 µm) and testis-removed fish (73.51 ± 9.18 µm) 2 months after surgery. A Sertoli cell-expressing Dmrt1 was used as a marker to identify the testicular tissue in the control (sham) fish and testis-removed fish. IHC staining results showed that Dmrt1 signals were observed in testicular tissue, and no signal was observed in ovarian tissue in the control (sham) fish (Figures 2J,K). Furthermore, no Dmrt1 signal was observed in ovarian tissue and connective tissue in the testis-removed fish (Figures 2L,M). To evaluate the status of gonadal tissue in the control (sham) fish and testis-removed fish, BrdU was used to label proliferating cells in this study. IHC staining results showed that the BrdU signal was mainly observed in the oogonia in the control (sham) fish (Figures 2N,O) and testis-removed fish (Figures 2P,Q). The proliferative activity of oogonia in control (sham) fish was significantly lower than that in the testis-removed fish (Figure 2S). BrdU signals were broadly observed in the testicular tissues of the control group, revealing that control (sham) fish entered the active male phase (Figure 2R). Taken together, our results showed that control (sham) fish and testis-removed fish had different oogonia proliferating activity 2 months after surgery.

### Pituitary signaling did not activate the female phase in testis removal-induced fish

**FIGURE 3:**
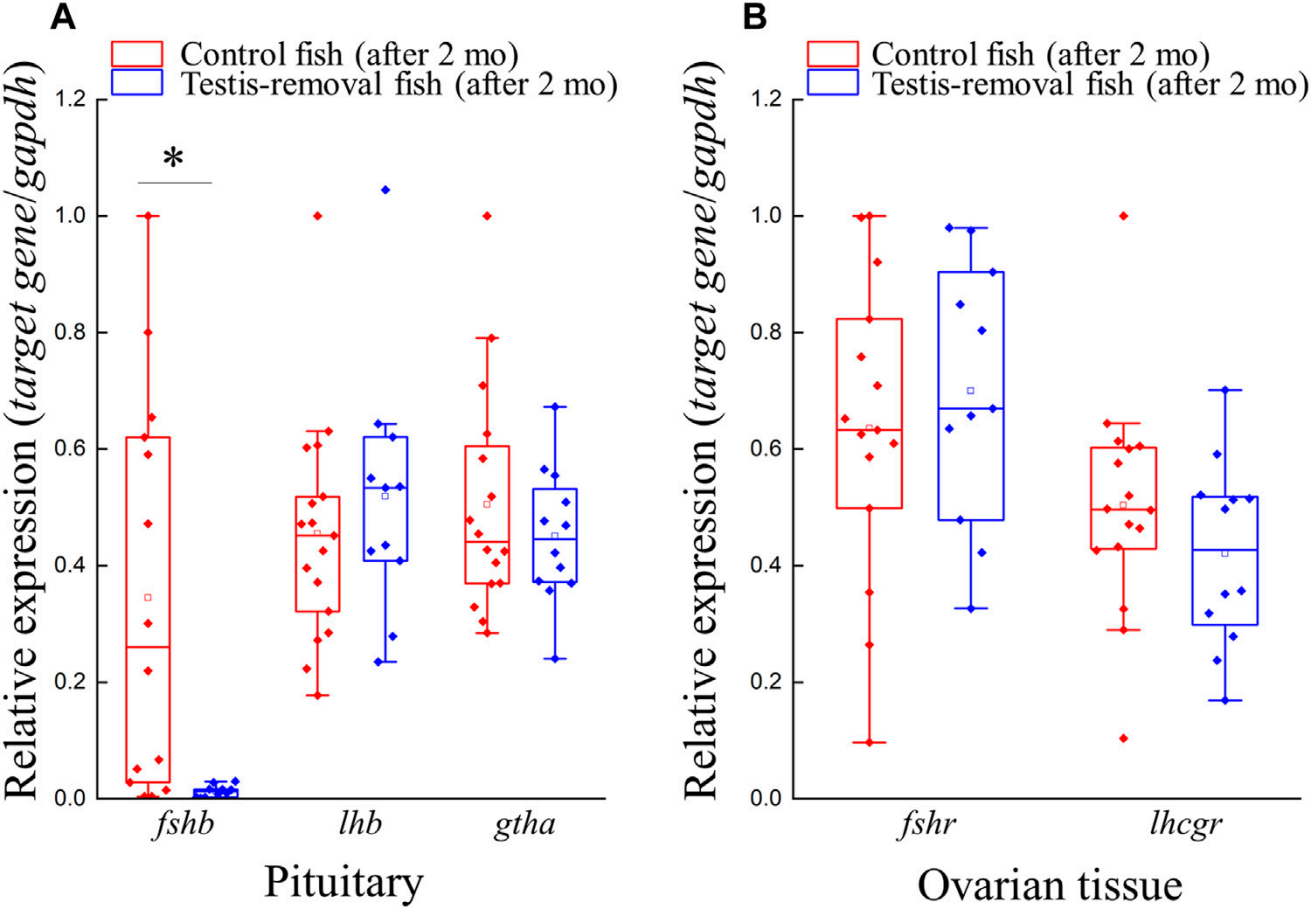
Genes expression profiles of gths (*fshb*, *lhb*, and *gtha*) and *gthrs* (*fshr* and *lhcgr*) after testis-removal induced femaleness. There was no difference in cell composition of ovarian tissue in the control fish and testis-removed fish at 2 months after surgery. To understand the change of *gths* and *gthrs* in secondary sex determination, qPCR analysis for pituitary and ovarian tissue was used, respectively. Relative expression was normalized with *gapdh*, and the highest sample value of each gene in the control fish was defined as 1. Asterisks indicate significant differences by Student’s *t*-test (*, p < 0.05).

Quantitative real-time PCR was performed on gonadotropin genes (*fshb*, *lhb*, and *gtha*) and their receptors (*fshr* and *lhcgr*) in pituitary and ovarian tissue, respectively. qPCR results showed that *fshb* transcripts in the pituitary were significantly decreased in the testis-removed fish compared to the control (sham) fish (Figure 3A). No difference in gene expression was found in *lhb* and *gtha* in the pituitary of either group (Figure 3A). In ovarian tissue, no difference in gene expression was found in the *fshr* and *lhcgr* of either group (Figure 3B). In the previous study, higher *lhcgr* and *fshr* transcripts were observed in testicular tissue compared to ovarian tissue at non- and pre-spawning season (Wu et al., 2016). Taken together, our data suggest that the female phase may not be activated by pituitary signaling in testis-removed black porgy.

### Gene expression of presumptive female-associated genes in testis removal-induced femaleness

**FIGURE 4:**
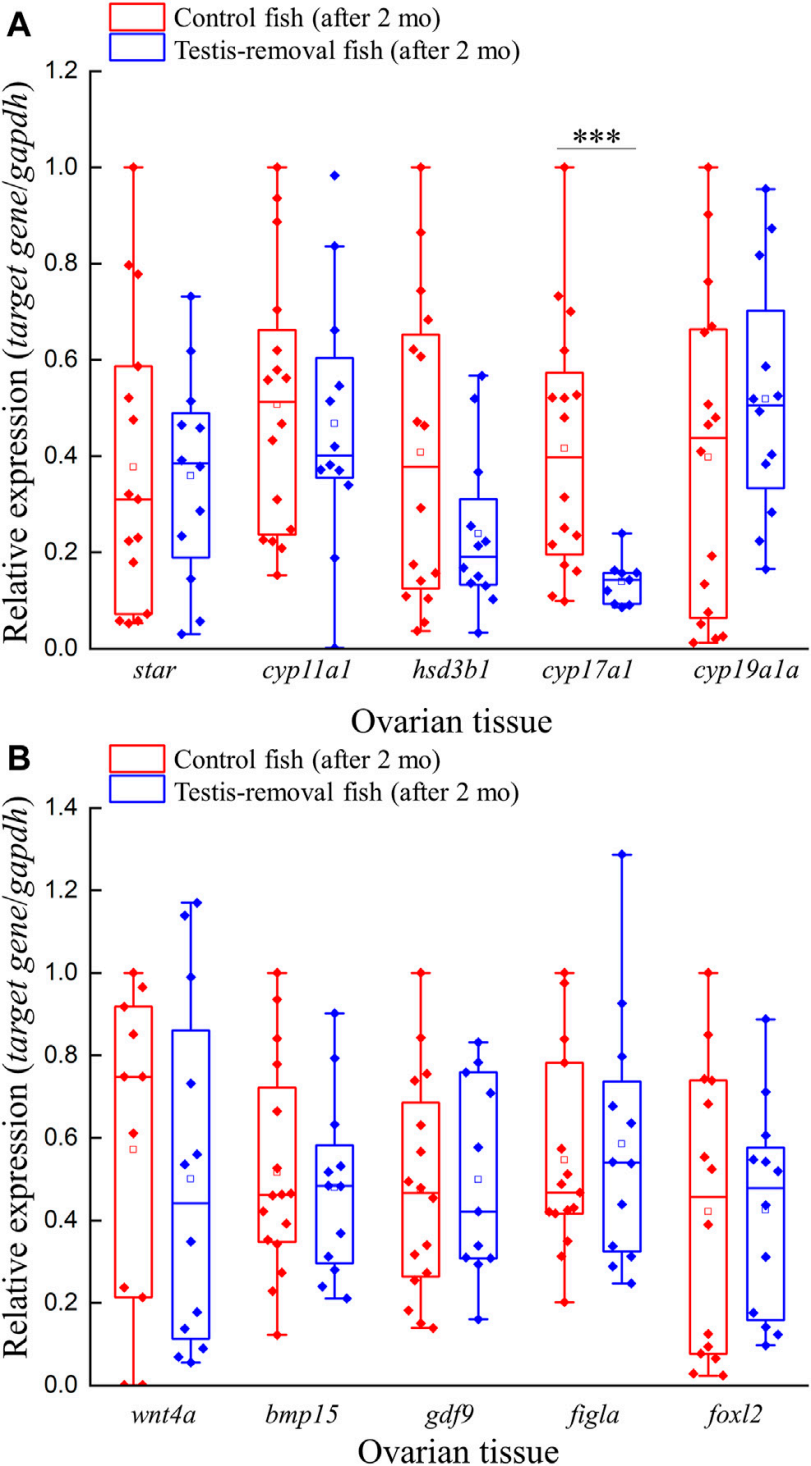
Expression profiles of ovarian steroidogenesis-related factors and female-related genes after testis-removal induced femaleness. To clarify the effect of sex steroids and female-related genes in secondary sex determination, we collected the ovarian tissue 2 months after surgery. The relative expression profiles of **(A)** ovarian steroidogenesis-related genes (*star*, *cyp11a1*, *hsd3b1*, *cyp17a1*, and *cyp19a1a*) and **(B)** potential female-related genes (*wnt4a*, *bmp15*, *gdf9*, *figla*, and *foxl2*) were detected using qPCR. Relative expression was normalized with *gapdh*, and the highest sample value of each gene in the control fish was defined as 1. Asterisks indicate significant differences by Student’s *t*-test (***, p < 0.001).

Quantitative real-time PCR was performed to evaluate the expression profiles of five steroidogenesis-related genes (*star*, *cyp11a1*, *hsd3b1*, *cyp17a1*, and *cyp19a1a*) and five female-related genes (*wnt4a*, *bmp15*, *gdf9*, *figla*, and *foxl2*) in ovarian tissue. qPCR results showed that ovarian *cyp17a1* transcripts were significantly decreased in the testis-removed fish compared to the control (sham) fish 2 months after surgery (Figure 4A). No difference in gene expression was found in other selected steroidogenesis-related genes (*star*, *cyp11a1*, *hsd3b1*, and *cyp19a1a*) in the ovarian tissue of either group 2 months after surgery (Figure 4A). Moreover, no difference in gene expression was found in selected female-related genes (*wnt4a*, *bmp15*, *gdf9*, *figla*, and *foxl2*) in the ovarian tissue of either group 2 months after surgery (Figure 4B). Thus, most female-associated genes were not involved in the early period of femaleness (male-to-female sex change) in testis-removed black porgy.

### RNA sequencing, *de novo* assembly, and functional annotation

RNA-seq was conducted on ovarian tissue of the control maleness and testis removal-induced female fish. A total of 304.8 million (92.1 Gb) 150-bp paired-end reads were generated that contained 172 million (52 Gb) and 132.8 million (40.1 Gb) reads in the control maleness and testis removal-induced femaleness groups, respectively (Table 1). After removal of low-quality reads and short read sequences, a total of 85.2 Gb reads (92.5%) were obtained from the control maleness (47.6 Gb, 91.5%) and testis removal-induced femaleness (37.6 Gb, 94%) groups, and then these reads were used for the following analysis (Table 1). Ovarian tissues from both groups were used to generate 190,992 transcripts and 128,453 unigenes (Table 2). The N50 value (the shortest contig that needs to be included for covering 50% of the assembled transcripts) of the unigenes was 1,069 bp (Table 2). Moreover, the E98N50 value (the topmost expressed genes which represent 98% of the total normalized expression data) was 2,405 bp, and E98 contained 15,864 unigenes from a total of 128,453 genes (Table 2 and Supplementary Figure S2). These unigenes contained 3,027 (83.2%) complete BUSCOs and 118 (3.2%) fragmented BUSCOs, while 495 (13.6%) BUSCOs were missing from 3,640 single-copy orthologs of actinopterygii (Table 2). Among these unigenes, 20,865, 12,703, 26,315, 27,130, and 27,597 genes were identified in the GO, KEGG, Pfam (a database of protein families), COG (a database of Clusters of Orthologous Genes), and eggNOG (evolutionary of genes: Non-supervised Orthologous Groups) databases, respectively (Table 3). The transcriptome data obtained from the samples were uploaded to the NCBI SRA site under BioProject accession number PRJNA767932.

**TABLE 1:** Summary of transcriptome data of black porgy (ovarian tissue from three control fish with ovotestis and three testis-removal fish)

Sample	Number of raw reads	Raw bases (G)	Number of clean reads	Clean bases (G)	CG content (%)
Control-1	74,747,107	22.6	74,744,826	20.6	52.3
Control-2	39,854,630	12.0	39,853,663	10.8	52.8
Control-3	57,487,857	17.4	57,486,210	16.2	52.8
Testis-removal-1	34,477,022	10.4	34,475,687	9.6	52.6
Testis-removal-2	60,510,035	18.3	60,506,823	17.2	52.6
Testis-removal-3	37,819,082	11.4	37,816,036	10.8	53.1

**TABLE 2:** Summary statistics of transcriptome assembly

Feature	Value
Number of genes (n)	128,453
Number of transcripts (n)	190,992
Total size of transcripts (bp)	176,432,272
Assembly GC content (%)	47.6
N50 (bp)	1,069
E98N50 (bp)	2405
Number of E98 (n)	15,864
Putative ORFs (TransDecoder) (n)	142,619
Complete BUSCOs (n)	3,027 (83.2%)
single-copy (n)	2,202 (60.5%)
duplicated (n)	825 (22.7%)
Fragmented BUSCOs (n)	118 (3.2%)
Missing BUSCOs (n)	495 (13.6%)

ORFs: Open reading frames; N50 is the shortest contig length that needs to be included for covering 50% of the assembled transcripts. In E98N50 statistic, we computed the N50 statistic that is limited to the topmost highly expressed genes which represent 98% of the total normalized expression data. In the present study, the maximum value is found in E98 that contains 15,864 genes from a total of 128,453 genes.

**TABLE 3:** Overview of functional annotation using eggNOG mapper v2

Database	Number of unigenes hits
eggNOG	27,597
Pfam	26,315
GO	20,865
KEGG	12,703
COG	27,130

eggNOG, evolutionary genealogy of genes: non-supervised orthologous Groups; Pfam, a database of Protein families; GO, gene ontology; KEGG, Kyoto Encyclopedia of Genes and Genomes; COG, database of clusters of orthologous genes.

### Differentially expressed genes between control maleness and testis removal-induced femaleness

**FIGURE 5:**
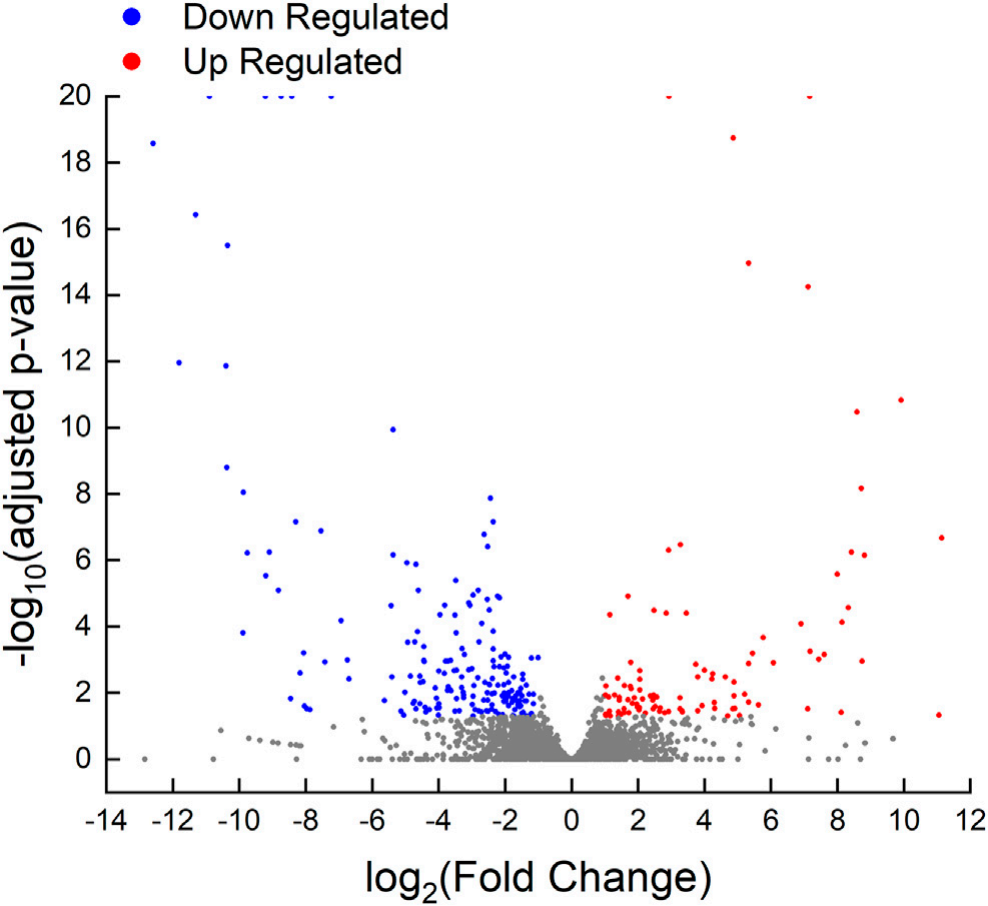
Volcano plot of differentially expressed genes in control fish and testis-removed fish. To find the genetic information during secondary sex determination, we performed differential expression analysis in the control fish and testis-removed fish with DESeq2. Genes with “adjusted p-value < 0.05” and “|log_2_Fold Change| > 1” were considered the DEGs (differentially expressed genes). The up-regulated (101 genes) and down-regulated (188 genes) DEGs in testis-removed fish were shown in red and blue dots, respectively. The gray dots represented the genes not belonging to DEGs.

A total of 289 unigenes were differentially expressed in the ovarian tissues between the control maleness and testis removal-induced femaleness group. In total, 101 unigenes were upregulated in testis removal-induced femaleness, and 188 genes were downregulated in the testis removal-induced femaleness compared with the control maleness group (Figure 5). According to the functions of the differentially expressed genes (DEGs), some upregulated genes in the testis-removed fish, such as *pex12*, *arg1*, and *stag3* genes, were related to reproduction (GO:0000003) and oocyte meiosis (ko04114). Some downregulated genes in the testis-removed fish, such as *ccnb2*, *pttg1*, and *epas1a*, participated in oogenesis (GO:0048477) and oocyte meiosis (ko04114). Thus, these DEGs may be involved in the process of femaleness (male-to-female sex change) in black porgy.

### Gene ontology and Kyoto Encyclopedia of Genes and Genomes analyses of differentially expressed genes in control maleness and testis removal-induced femaleness

**FIGURE 6:**
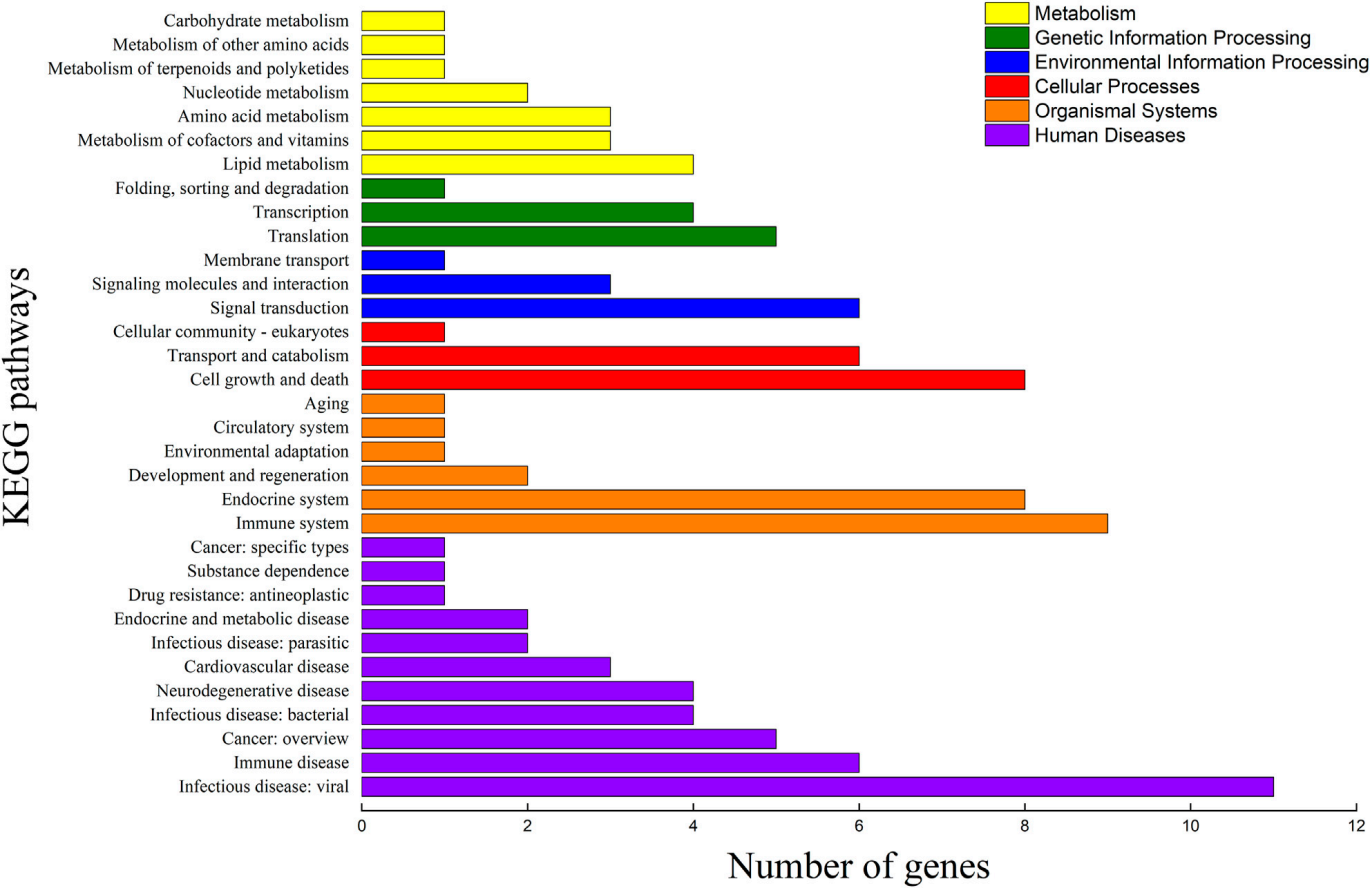
KEGG pathway classification of DEGs. The x-axis indicates the number of DEGs (differentially expressed genes) matched in KEGG (Kyoto Encyclopedia of Genes and Genomes) pathway. The y-axis indicates the 33 KEGG subcategories.

**FIGURE 7:**
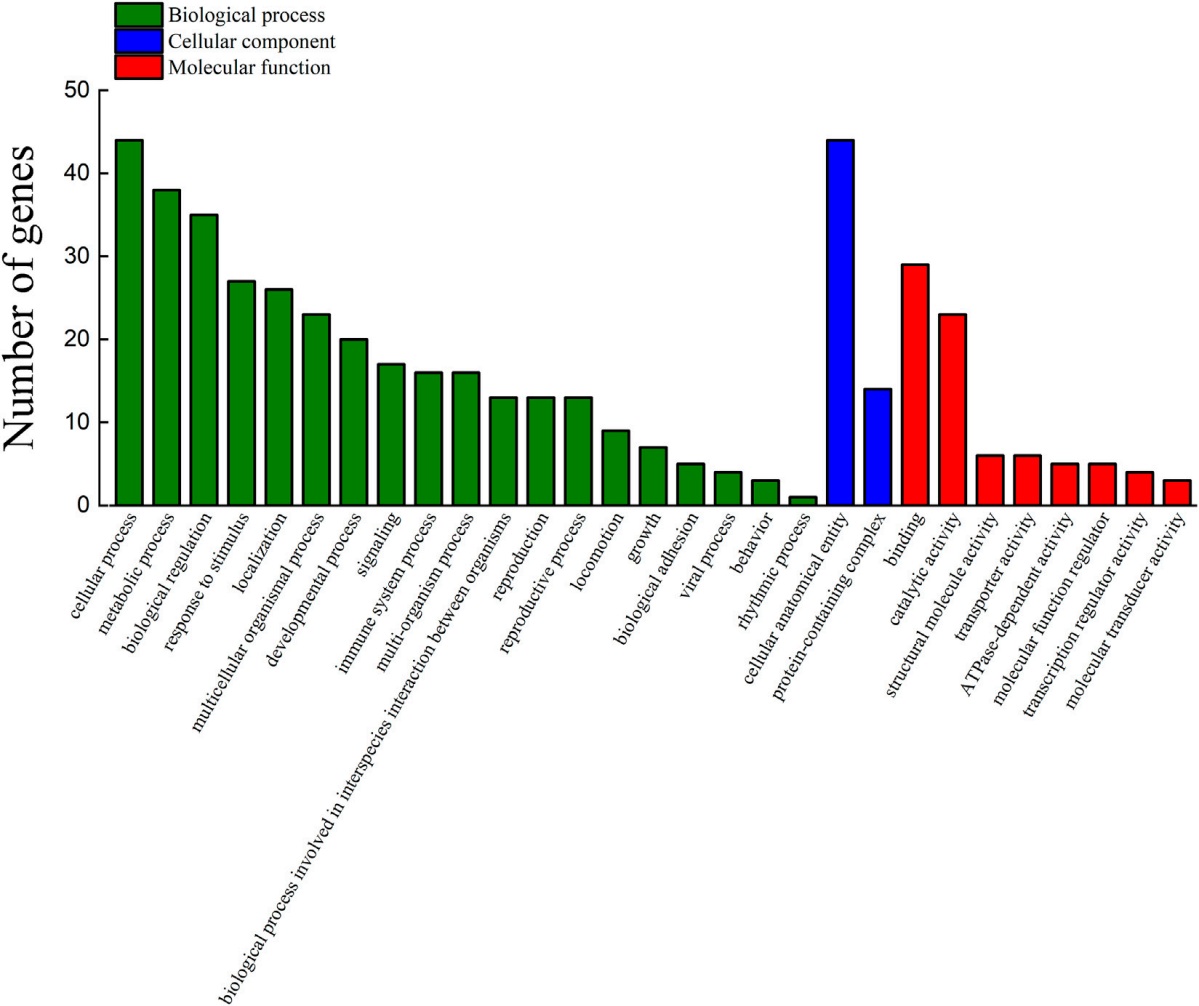
Functional annotation of DEGs in the GO database. The counts of DEGs (differentially expressed genes) matched in level 2 GO (Gene Ontology) categories. The x-axis indicates 29 levels 2 GO categories. The y-axis indicates the number of DEGs that were matched.

Among the 289 DEGs, 55 and 45 DEGs were matched in the KEGG and GO databases, respectively. However, 234 (81%) DEGs were not matched in any databases. The results showed that 55 and 45 DEGs were annotated to 33 KEGG subcategories and 29 level 2 GO categories, respectively (Figures 6, 7). In GO, 2, 9, and 2, DEGs were annotated as oogenesis (GO:0048477), reproduction (GO:0000003), and meiosis I cell cycle process (GO:0061982), respectively (Table 4). At the same time, 3 DEGs were matched to oocyte meiosis (ko04114) in the KEGG database (Table 4). Taken together, our results demonstrated that increased oogonia proliferation in testis-removed protandrous black porgy was an important event in secondary sex determination, and we identified several candidate genes that could be involved in this process. In addition to the above genes, many unknown DEGs that we identified could have important roles in secondary sex determination.

**TABLE 4:** The candidate DEGs (differentially expressed genes) involved in GO (Gene Ontology) terms and KEGG (Kyoto Encyclopedia of Genes and Genomes) pathway. log_2_FC = log_2_ fold change.

GO term/KEGG pathway	Gene	DESeq2 base mean		log_2_FC	adjusted p-value
		Control fish (maleness)	Testis-removed fish (femaleness)		
Oogenesis (GO:0048477)	*epas1*	391	2	−7.42	1.17E-03
	*hnrnpa1*	576	5	−6.94	6.74E-05
reproduction (GO:0000003)	*ptk7*	122	36	−1.74	4.94E-02
	*ncam1*	382	66	−2.53	9.94E-03
	*pex12*	116	315	1.44	1.38E-02
	*arg1*	1,477	4785	1.70	1.24E-05
	*lnpep*	630	56	−3.50	4.51E-05
	*plcd3*	293	2	−7.23	6.66E-24
	*ccnb2*	2795	167	−4.07	1.46E-02
	*cdk16*	208	9	−4.57	3.16E-03
	*junb*	177	70	−1.35	5.84E-03
Meiosis I cell cycle process (GO:0061982)	*lmna*	104	0	−9.09	5.77E-07
	*pttg1*	31878	13774	−1.21	9.06E-04
Oocyte meiosis (ko04114)	*ccnb2*	2795	167	−4.07	1.46E-02
	*pttg1*	31878	13774	−1.21	9.06E-04
	*stag3*	988	344	−1.52	7.36E-03

### Validation of differentially expressed genes by quantitative real-time PCR

**FIGURE 8:**
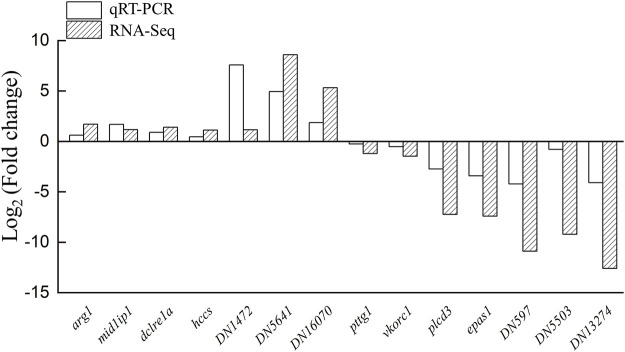
Validation of transcriptomic data by qPCR. The 14 DEGs (differentially expressed genes) were selected randomly to detect the expression profiles. Some genes were not matched in any databases, and we named these genes DN1472, DN5641, DN16070, DN597, DN5503, and DN13274. The qPCR results were consistent with the transcriptomic data. y-Axis indicates Log_2_ (Gene expression profile of testis-removed fish/Gene expression profile of control fish).

To confirm the reliability of the transcriptomic analysis, 14 DEGs were detected by qRT-PCR randomly. qPCR analyses indicated that the expression profiles of *arg1*, *mid1ip1*, *dclre1a*, *hccs*, *DN1472*, *DN5641*, and *DN16070* were increased in the testis-removed fish compared to the control (sham) fish. Meanwhile, the expression profiles of *pttg1*, *vkorc1*, *plcd3*, *epas1*, *DN597*, *DN5503*, and *DN13274* were decreased in the testis-removed fish compared to the control (sham) fish (Figure 8). These qPCR results were consistent with the transcriptomic data.

## Discussion

Unlike the reversible sex change (female-to-male) found in long-term E_2_-treated fish after E_2_ withdrawal, precocious femaleness (oocytes entering the vitellogenic oocyte stage and/or maturation) was observed in testis-removed fish in <2-year-old black porgy. This characteristic was applied to study the molecular mechanism of secondary sex determination/female phase. Cell proliferation analysis showed that higher oogonia proliferation was found in testis removal-induced femaleness compared to control (sham) maleness. Most female-associated genes found in previous studies had no difference in the early status of testis removal-induced femaleness compared to the control (sham) maleness 1 and 2 months after surgery. A small number of genes (289 genes) showed dimorphic expression of sexual fate in both groups by transcriptomic analysis. Our data identified that the first characteristic of femaleness was a large number of proliferating germline cells in the testis-removed female compared to the control (sham) maleness 2 months after surgery. Our results further identified several candidate genes in ovarian tissue that may be involved in the early period of secondary sex determination (femaleness) in black porgy.

### Testis removal induced the oogonia proliferation and further development in the ovotestis

Oogonia proliferation is necessary for ovary development in teleosts and is the basis for oogenesis (Nakamura et al., 2010). In this study, our data showed that testis removal-induced femaleness had higher oogonia proliferating activity compared to the control (sham) maleness 2 months after surgery in <2-year-old fish (Figure 2S). This is the important sign in testis removal-induced sex change during the early period. In order to find the key and earlier genetic factors involved in the early sex change (even before 2 months), we used the transcriptomic analysis of the ovarian tissue 1 month after the surgery of testis removal.

In our previous study, IHC staining of Pcna showed that Pcna signals were broadly observed in oogonia of ovarian tissue and that fewer Pcna signals were observed in spermatogonia of testicular tissue during the post- and non-spawning seasons (status 6 and 7, developed ovarian tissue with regressed testicular tissue) in <2-year-old maleness (Wu et al., 2010a, Wu et al., 2015). This gonadal status was altered from female to male (status 8, developed testicular tissue with regressed ovarian tissue) during the prespawning season. Pcna signals were broadly observed in spermatogonia of testicular tissue, and fewer Pcna signals were observed in oogonia of ovarian tissue during the prespawning season in <2-year-old maleness (Wu et al., 2010a, Wu et al., 2015). Taken together, these data demonstrated that testis-removed fish had the characteristic of female fate (femaleness) 2 months after surgery. These results also demonstrate that the developing testicular tissue inhibits oogenesis (ovarian growth) in the male fate (maleness) of black porgy.

### Differential response of Gths signaling in active maleness and passive femaleness in black porgy

Gonadotropins can activate the gonad *via* their receptors to regulate gametogenesis and gonadal development in fish. In this study, the gene expression profile of gonadotropins in the pituitary showed differences between control (sham) maleness and testis removal-induced femaleness (Figure 3A). Two months after surgery, a lower gene expression level of *fshb* in the pituitary was found in testis removal-induced femaleness compared to control (sham) maleness, and no difference in *lhb* and *gtha* transcripts was found in either group (Figure 3A). In an *in vitro* testicular culture, recombinant Fsh induced the proliferation of spermatogonia and their spermatogenesis in Japanese eel (*Anguilla japonica*) (Ohta et al., 2007). In an *in vivo* injection, recombinant Fsh caused an increase in spermatogonia proliferation in European sea bass (*Dicentrarchus labrax*) (Mazón et al., 2014). Thus, the high level of Fsh plays important roles in spermatogenesis through the stimulation of 11-KT production in fish. In hermaphroditic fish, precocious maleness (female-to-male sex change) can be induced by the administration of recombinant Fsh in the protogynous honeycomb grouper (*Epinephelus merra*) (Kobayashi et al., 2010) and the protogynous giant grouper (*Epinephelus fuscoguttatus*) (Palma et al., 2019). In contrast, precocious maleness was not induced by exogenous recombinant Lh in the protogynous honeycomb grouper (Kobayashi et al., 2010). Furthermore, precocious femaleness was not induced by a luteinizing hormone-releasing hormone analog (LHRHa) and hCG in black porgy (Chang et al., 1995b; Du et al., 2005). We also found that plasma Lh levels were higher in the males compared to the naturally sex-changing fish (from male to female) in 2–3-year-old black porgy (Du et al., 2005) and were also increased in the reversible sex change (from E_2_-induced female to male after 2 months of terminated E_2_ administration) in 1-year-old black porgy (Lee et al., 2004). Our present studies in pituitary *fshb* and previous data in plasma Lh (Lee et al., 2004; Du et al., 2005) may support the importance of gonadotropins in the male phase of black porgy. Thus, this decrease in pituitary *fshb* transcript levels in testis removal-induced femaleness may be associated with the sexual fate of brain-pituitary signaling in protandrous black porgy.

In the protandrous clownfish (*Amphiprion melanopus*), the gene expression levels of *fshr* and *lhcgr* were significantly increased in syngonic gonads during the male-to-female sex change (An et al., 2010). In gobiid fish (*Trimma okinawae*) with bidirectional sex changes, sex reversal is mediated by *G*th receptors in the active gonad, which expresses high levels of *fshr* and *lhcgr* (Kobayashi et al., 2009). In protandrous gilthead sea bream (*Sparus aurata*), the gene expression patterns of gthrs are dimorphic in the digonic gonad, with the testis showing high *fshr* expression and the ovary showing high *lhcgr* expression (Wong and Zohar 2003). In this study (Figure 3B) and our previous study (Wu et al., 2016), no differences in *fshr* and *lhcgr* expression in ovarian tissue were found at the early developmental stage (status 7, nonspawning season) in testis removal-induced femaleness compared to control (sham) maleness. Moreover, increased gene expression levels of *fshr* and *lhcgr* in testicular tissue were associated with the sexual phase from passive femaleness to maleness during the nonspawning season (status 7) in <2-year-old black porgy (Wu et al., 2016). Furthermore, our previous study showed that testicular *dmrt1* expression is stimulated by *in vivo* LHRH analog treatment or *in vitro* hCG treatment (Wu et al., 2012). *Dmrt1* is an important gene involved in testicular differentiation and development in black porgy, and a regressed testis and developed ovary were found in *dmrt1*-deficient juvenile black porgy (Wu et al., 2012). Taken together, our data demonstrated that the hypothalamus-pituitary-testis axis and testicular *dmrt1* are important for male fate maintenance in black porgy (Wu and Chang, 2018; Wu et al., 2021).

### First transcriptomic analysis at the tissue level of the ovotestis in hermaphroditic fishes

In this study, transcriptomic analysis of ovarian tissue separated from the ovotestis showed 289 DEGs that may be involved in testis removal-induced femaleness in protandrous black porgy. To date, the molecular mechanism of sex change in several hermaphroditic fish has been studied through transcriptomic analysis. Based on these studies, a large number of DEGs were found between the different gonadal statuses, including the digonic species, such as 22,449 DEGs in the protandrous black porgy with a digonic gonad (Zhang et al., 2019), 22,591 DEGs in the yellowfin seabream with a digonic gonad (*Acanthopagrus latus*) (Li et al., 2020), 25,928 DEGs in the protogynous common pandora with a digonic gonad (*Pagellus erythrinus*) (Tsakogiannis et al., 2018), 18,724 DEGs in the juvenile bisexually red porgy with a digonic gonad (*Pagrus*) (Tsakogiannis et al., 2018), and syngonic species, such as 5,483 DEGs in the protogynous clownfish with a syngonic gonad (*Amphiprion bicinctus*) (Casas et al., 2016) and 20,183 DEGs in the protogynous bluehead wrasse (*Thalassoma bifasciatum*) (Liu et al., 2015). These positive results (i.e. the large number of DEGs found in these hermaphroditic fish studies) may be due to the transcriptomic studies performed on the whole ovotestis, which is not a separated ovarian and testicular tissue of the ovotestis. Thus, similar results were found in gonochoristic and hermaphroditic fish species. The gonadal transcriptomic results in gonochoristic fishes showed that approximately 6,000–15,000 DEGs were found between the sexes, such as Nile tilapia (Tao et al., 2013), yellow catfish (*Pelteobagrus fulvidraco*) (Lu et al., 2014), olive flounder (*Paralichthys olivaceus*) (Fan et al., 2014), and golden pompano (*Trachinotus ovatus*) (He et al., 2020). In hermaphroditic fish, the relative proportions of testicular and ovarian tissue are dynamic during sex fate alternation. The gene expression profiles may be affected by the ratio of testicular versus ovarian tissues and are difficult and inaccurate to analyze due to the reciprocal gonad tissues mixing together. By applying the separation of the testicular and ovarian tissues of the ovotestis in this study, our approach (by the comparison of ovarian tissues collected from maleness and testis removal-induced femaleness) could enhance the resolution of the gonadal transcriptome and then identify the key factors in the process of sex fate alternation in hermaphroditic fishes.

### Potential factors may regulate secondary sex determination in hermaphroditic fish

**FIGURE 9:**
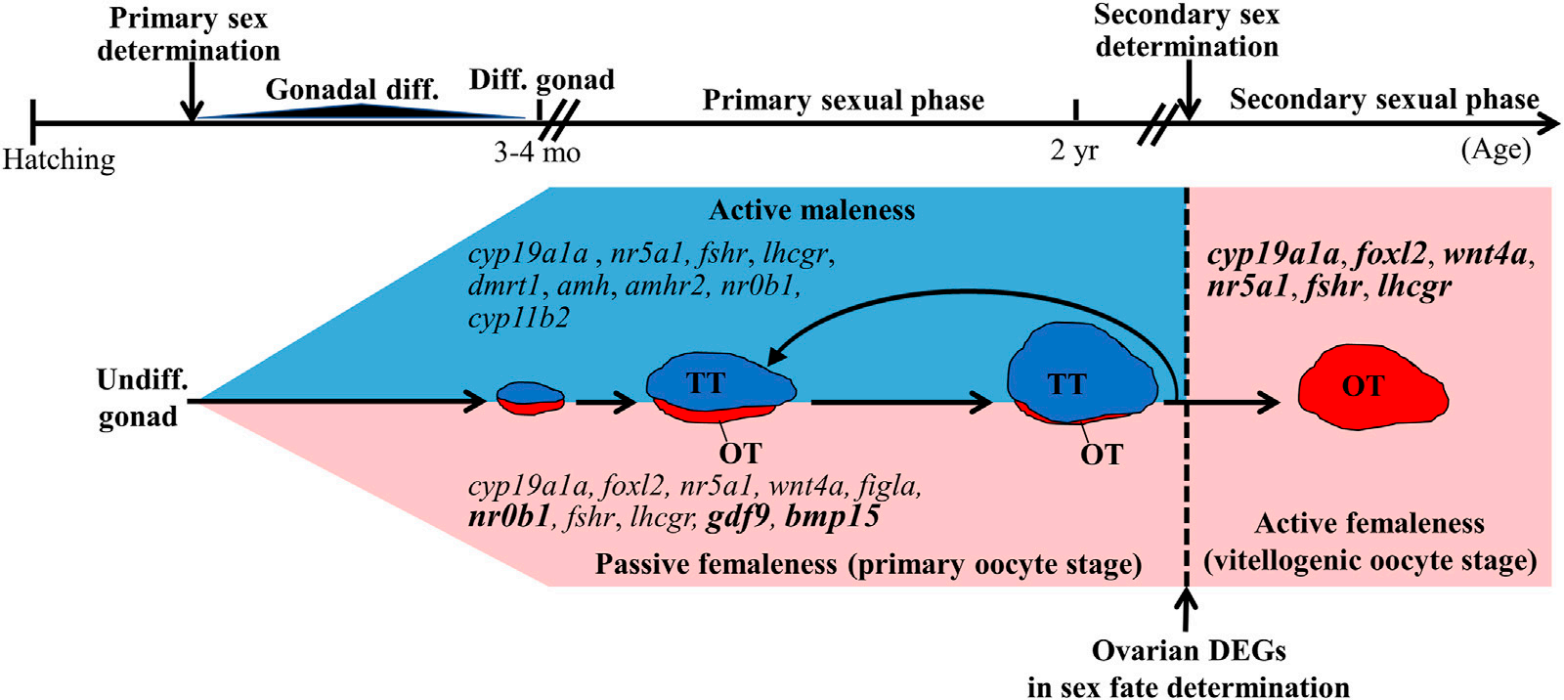
Summary of sex-related genes involved in sex determination, differentiation, and development in black porgy. Primary sex determination and gonadal differentiation occurred around 3–4 months after hatching. But the exact timing and genes of primary sex determination are still not clear. The fish has functional males during the first two reproductive cycles. After secondary sex determination, we can observe developing ovaries with rudimentary testis. However, the key factors that activate secondary sex determination are still not clear. DEGs (differentially expressed genes) found in this study may be a potential secondary sex determination gene. Bold letters in the pink area indicate the genes have significantly higher expressions in the ovarian tissue with primary oocyte stage compared with vitellogenic oocyte stage. Genes in the pink area indicate that the genes expressed at high levels in the ovary. Genes in the blue area indicate that the genes expressed at high levels in the testis. Undiff. gonad, undifferentiated gonad; Diff. gonad, differentiated gonad; Gonadal diff., Gonadal differentiating.

In sequential hermaphroditic fishes, the timing for secondary sex determination is determined by age or body size. However, the factors that activate secondary sex determination are still not clear. In this study, we found 289 DEGs from the ovarian tissues between male (control fish) and potential female (testis-removed fish) during the secondary sex determination. Functional annotation showed that several DEGs were matched in oogenesis (GO:0048447), meiosis I cell cycle process (GO:0061982), and oocyte meiosis pathways (ko04114), such as *stag3* (Prieto et al., 2001), *ccnb2* (Han et al., 2017; Daldello et al., 2019), and *pttg1* (Terret et al., 2003), which have been identified as mammalian oogenesis-related genes (Table 4). These results are consistent with the cell proliferation assay, which showed decreased oogenesis in control fish (with developing testis). However, 81% of DEGs were still not matched in online databases in the present study. In a previous study, the transcriptomes of some hermaphroditic fish were also evaluated to identify the sex determination genes. A previous transcriptomic study on black porgy gonads provided several candidate genes (*jnk1*, *vasa*, *wnt4*, *figla*, and *foxl2*) that are associated with secondary sex determination and differentiation (Zhang et al., 2019). The steroidogenesis-related genes (such as *hsd17b1*, *hsd11b3*, *hsd17b12*, and *cyp19a1a*), androgen/estrogen receptor genes (such as *ara*, *arb*, and *erb*), Wnt/beta-catenin signaling genes (such as *wnt4a*, *wnt9b*, *ctnnb1*, and *fst*), and female-related genes (such as *foxl2*, *gdf9*, *bmp15*, *gsdf*, and *sox3*) are considered to involve female sex fate in yellowfin seabream (Li et al., 2020), bluehead wrasses (Liu et al., 2015), ricefield eel (Cai et al., 2017), common pandora (Tsakogiannis et al., 2018), and red porgy (Tsakogiannis et al., 2018) (Supplementary Table S2). Although some candidate genes in female fate were found in many teleosts through the transcriptome, those genes were not among the DEGs that were analyzed in this study (Supplementary Table S3), possibly due to sample selection (i.e. cell population or stage in gonads). These results indicate that many candidate genes may not be female sex determination genes but ovarian differentiation and development genes. This study provides several potential factors that may regulate secondary sex determination in hermaphroditic fish (Figure 9).

## Conclusion

We studied secondary sex determination using testis removal-induced femaleness in <2-year-old black porgy. The results demonstrated that oogonia proliferation activity is the first sign of secondary sex determination. Furthermore, Gth-Gthr signaling may not cause secondary sex determination. Our ovarian transcriptomic study and qPCR analysis showed that female-related genes in black porgy such as *wnt4a* (Wu and Chang, 2009; Wu et al., 2017), *figla* (Wu et al., 2008b), *foxl2* (Wu et al., 2008b, Wu et al., 2016), *gdf9* (Wu et al., 2017), *bmp15* (Wu et al., 2017), and *cyp19a1a* (Wu et al., 2008b) were not involved in the early phase of femaleness (Figure 9). To the best of our knowledge, this is the first transcriptomic study to compare ovarian tissue between different sexual fates (maleness and femaleness) in hermaphroditic fish. We identified a few sex-related DEGs that matched in reproduction (GO:0000003), oogenesis (GO:0048477), and oocyte meiosis (ko04114). Furthermore, the functions of 234 (81%) DEGs are still unknown. The potential roles of these DEGs should be further examined with regard to the sex fate alternation of the ovarian tissue.

## Data Availability

The original contributions presented in the study are included in the article/Supplementary Material, further inquiries can be directed to the corresponding authors.
