# Supplementary figures and images for: The Ovarian Transcriptome at the Early Stage of Testis Removal-Induced Male-To-Female Sex Change in the Protandrous Black Porgy Acanthopagrus schlegelii
Source: Front Genet. 2022 Mar 23;13:816955. doi: 10.3389/fgene.2022.816955 (PMC8986339; doi:10.3389/fgene.2022.816955)

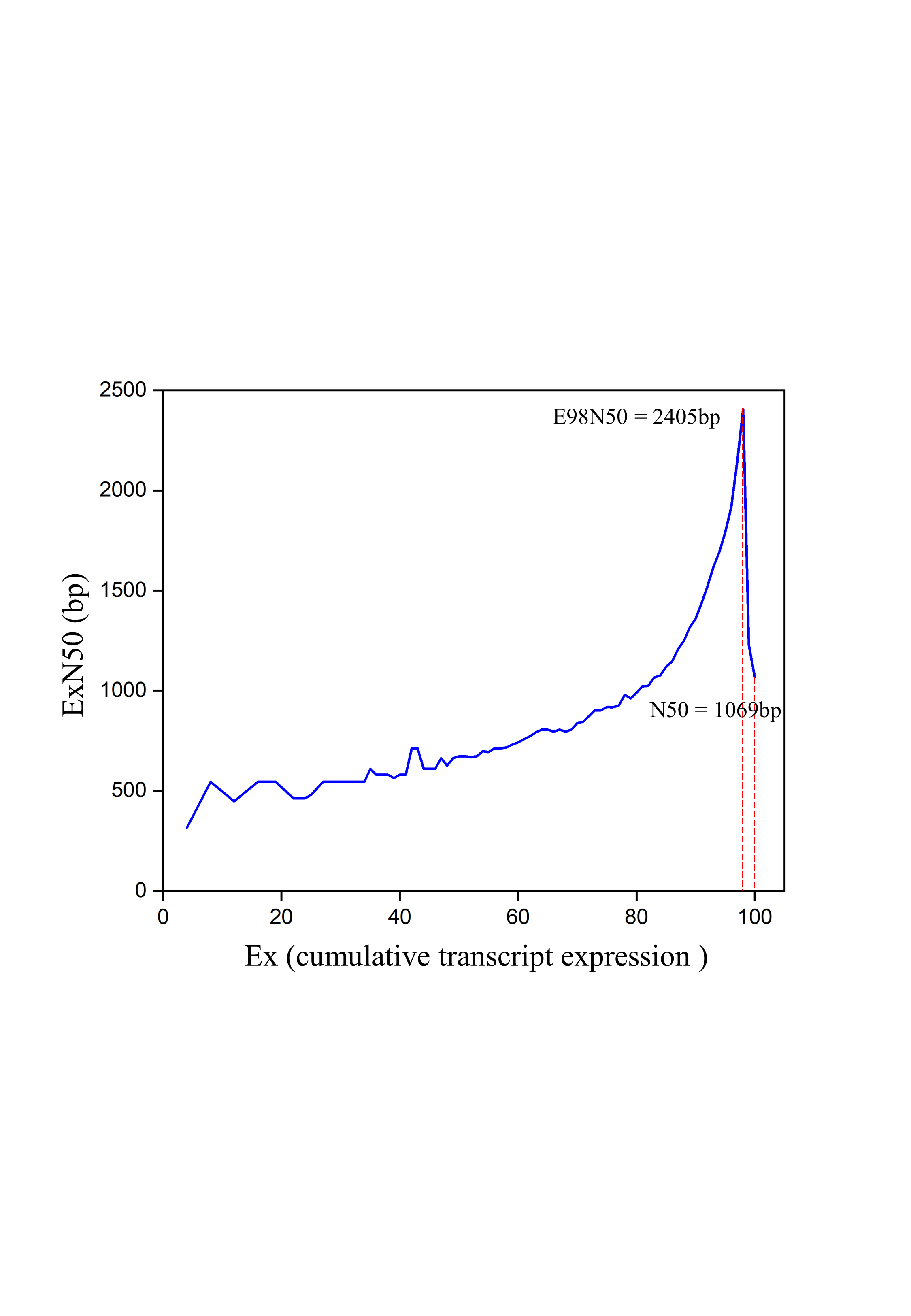

Supplement: Supplementary file 1 [file Image2.TIF]

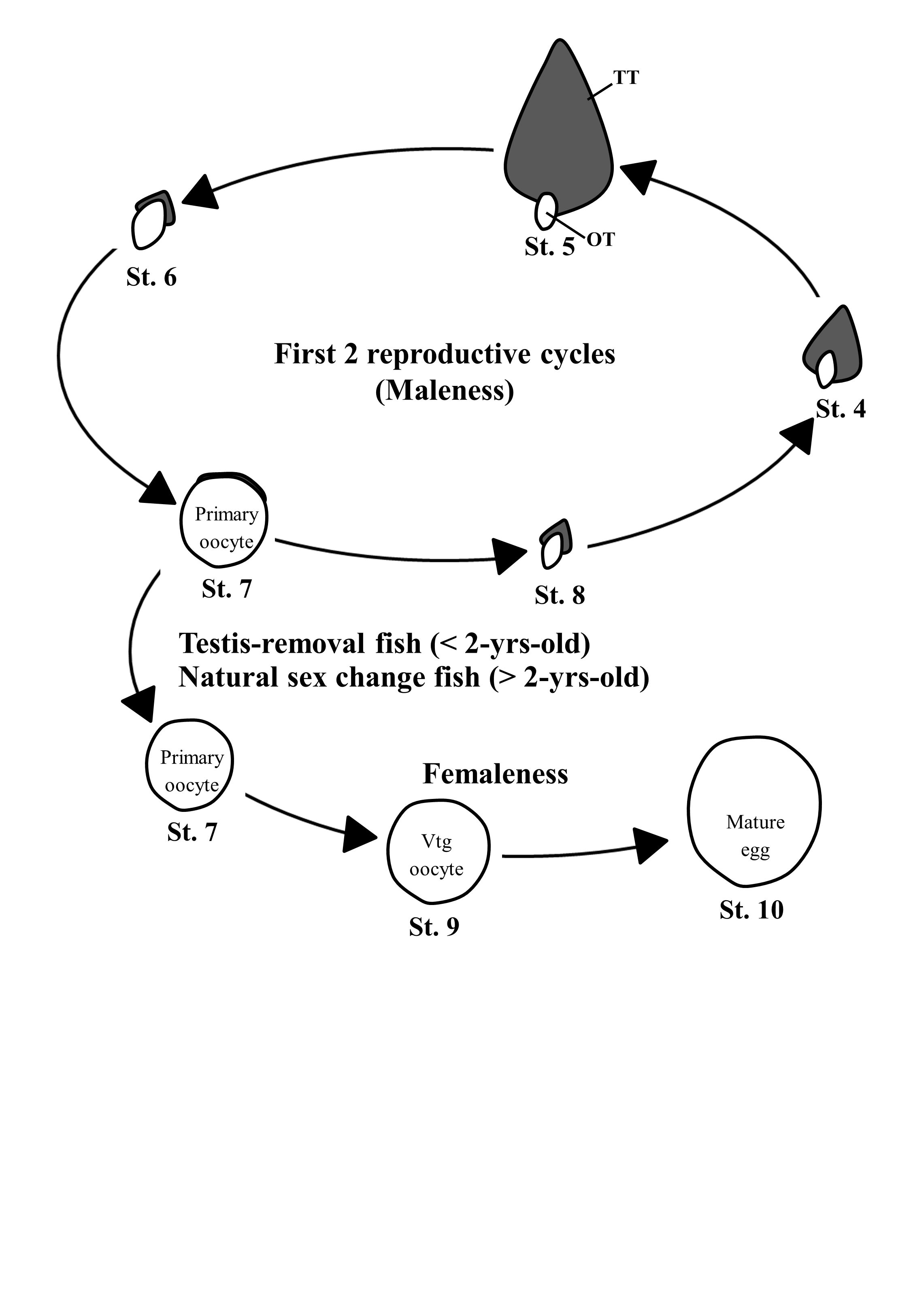

Supplement: Supplementary file 2 [file Image1.TIF]
