# Supplementary material for: The Ovarian Transcriptome at the Early Stage of Testis Removal-Induced Male-To-Female Sex Change in the Protandrous Black Porgy Acanthopagrus schlegelii
Source: Front Genet. 2022 Mar 23;13:816955. doi: 10.3389/fgene.2022.816955 (PMC8986339; doi:10.3389/fgene.2022.816955)
Supplement: Supplementary file 3 [file DataSheet1.docx]

**Supplemental Figure Legends**

**Supplementary Figure 1. Schematic diagram of the gonadal status in black porgy**. The fish are maleness in the first two reproductive cycles and the digonic gonads are separated by connective tissue. The male-to-female sex change occurred after surgically removing the testis. The gonadal status was divided as follows: developed testes with spermatocytes (status 4), developed testes with spermatozoa (status 5), regressing testes with an active ovary (status 6), dominant ovary with regressed testes (status 7), regressing ovaries with active testes (status 8), dominant ovary and oocytes with vitellogenesis (status 9), and functional female with rudimentary testis (status 10). St., gonadal status; Vtg oocyte, vitellogenic oocyte; TT, testicular tissue; OT, ovarian tissue.

**Supplementary Figure 2. Ex98N50 statistic and Ex98 gene count.** The quality assessment of *de novo* assembled transcriptome by computing ExN50 statistic. The X-axis (Ex) indicates the cumulative transcript expression. The Y-axis (ExN50) indicates the N50 in different cumulative transcript expressions. The original N50 value is 1069 bp and the E98N50 has a maximum N50 value. The E98N50 value is 2405 bp and E98 contains 15864 genes from a total of 128,453 genes.

**Supplementary Table 1.** Specific primers were used for qPCR analysis.

| Gene | Orientation | Sequence |
| --- | --- | --- |
| *gapdh* | Sense | 5′-AGGCTTCCTTAATCTCAGCATAAGAT-3′ |
|  | Antisense | 5′-GGTGCCTGTGGCTGATGTG-3′ |
| *fshb* | Sense | 5′-GATTCGGCTGCCATCCAA-3′ |
|  | Antisense | 5′-TGCACATATGGTGGTGTCAATG-3′ |
| *lhb* | Sense | 5′-GAGAAAGAAGGATGTCCCAAGTGT-3′ |
|  | Antisense | 5′-CTGGGTCCTTGGTGATGCA-3′ |
| *gtha* | Sense | 5′-CCCGAAGAACATCACCTCTGA-3′ |
|  | Antisense | 5′-TATGCCGGCCACCTCTGT-3′ |
| *star* | Sense | 5′-GCTCCCTTCTCAGCTCTCGTATTAG-3′ |
|  | Antisense | 5′-TCCTCGCCTTGCTTCACATAA-3′ |
| *cyp11a1* | Sense | 5′-GACACATTGGAGCGAAAGTTTG-3′ |
|  | Antisense | 5′-AGCGGTCCGCTTGACTGA-3′ |
| *hsd3b1* | Sense | 5′-GCTTCTGGAGGCATGTATTCAAG-3′ |
|  | Antisense | 5′-TTGGTCCCAGCACCTCTATTG-3′ |
| *cyp17a1* | Sense | 5′-AGTGAGGAAAGGAACTCGGGTTA-3′ |
|  | Antisense | 5′-TCAGGGTTTTCCCATTCTTTCTC-3′ |
| *cyp19a1a* | Sense | 5′-ACAAACCCGACGAATTCAGACT-3′ |
|  | Antisense | 5′-CCCGAACGGCTGGAAGTA-3′ |
| *wnt4a* | Sense | 5′-GGAGACAATGCGAGAGGTTACG-3′ |
|  | Antisense | 5′-TCACCTCCACACTGCGCTTA-3′ |
| *bmp15* | Sense | 5'-GCTACCATTCCCCCAACCA-3' |
|  | Antisense | 5'-CGCCCAGGTCGTTGATG-3' |
| *gdf9* | Sense | 5'-GACCAGAAGAGCAGAAGGAACTG-3' |
|  | Antisense | 5'-CATCAAGAGAGGCCGAAGAAA-3' |
| *figla* | Sense | 5′-CAGGAACTTGAACACCATGTTCTC-3′ |
|  | Antisense | 5′-CTTACGGTCTGGTCGCATTAGTG-3′ |
| *foxl2* | Sense | 5′-CAGGAACTTGAACACCATGTTCTC-3′ |
|  | Antisense | 5′-CTTACGGTCTGGTCGCATTAGTG-3′ |
| *fshr* | Sense | 5′-CACCCTGGAGCGCTGGTA-3′ |
|  | Antisense | 5′-CGTGTCTCAGGCGAAGTTTG-3′ |
| *lhcgr* | Sense | 5′-CGTCACGGAGACACCAAGATC-3′ |
|  | Antisense | 5′-CCATACACACAAAGTCGGTGAAA-3′ |

**Supplementary Table 2.** The genes are considered to involve teleost female sex fate by transcriptomic analysis.

|  | Gene | Yellowfin seabream | Bluehead wrasse | Ricefield eel | Common pandora | Red porgy |
| --- | --- | --- | --- | --- | --- | --- |
| steroidogenesis-related genes | *cyp19a1a* | **+** | **+** | **+** | **+** | **+** |
|  | *hsd17b1* | **+** | **+** |  | **+** | **+** |
|  | *hsd17b12* | **+** |  |  |  |  |
|  | *hsd11b3* |  | **+** |  |  | **+** |
| androgen/estrogen receptor genes | *ara* |  |  |  | **+** | **+** |
|  | *arb* |  |  |  | **+** | **+** |
|  | *erb* |  |  | **+** |  |  |
| Wnt/beta-catenin signaling | *wnt4a* | **+** | **+** |  | **+** | **+** |
|  | *wnt5a* | **+** |  | **+** |  |  |
|  | *wnt9b* | **+** |  | **+** |  |  |
|  | *wnt11* |  |  | **+** |  |  |
|  | *wnt16* | **+** |  |  |  |  |
|  | *axin2* | **+** |  |  |  |  |
|  | *lef1* | **+** |  |  |  |  |
|  | *rspo3* |  |  | **+** |  |  |
|  | *ctnnb1* |  | **+** | **+** | **+** | **+** |
|  | *fst* |  |  |  | **+** | **+** |
|  | *fstl4* |  | **+** |  |  |  |
| retinoid acid signaling pathway | *aldh1a2* |  |  | **+** |  |  |
|  | *cyp26a1* |  |  | **+** |  |  |
|  | *cyp26b1* |  |  | **+** |  |  |
|  | *cyp26c1* |  |  | **+** |  |  |
|  | *rdh3r* |  |  | **+** |  |  |
|  | *rdh5* |  |  | **+** |  |  |
|  | *rdh8* |  |  | **+** |  |  |
|  | *rdh12* |  |  | **+** |  |  |
| female-related gene | *gdf9* |  | **+** | **+** | **+** | **+** |
|  | *bmp15* |  |  | **+** |  |  |
|  | *gsdf* |  |  |  |  | **+** |
|  | *foxl2* | **+** | **+** | **+** | **+** | **+** |
|  | *dmrt5* | **+** |  |  |  |  |
|  | *sox3* | **+** | **+** |  | **+** | **+** |
|  | *sox2* |  | **+** |  |  |  |
|  | *wt1a* |  |  |  | **+** | **+** |
|  | *wt1a* |  |  |  | **+** | **+** |

+ means the gene is considered to be related to female fate in yellowfin seabream, *Acanthopagrus latus* (Li et al., 2020), bluehead wrasse, *Thalassoma bifasciatum* (Liu et al., 2015), ricefield eel, *Monoptrus albus* (Cai et al., 2017), common Pandora, *Pagellus erythrinus* (Tsakogiannis et al., 2018), and red porgy, *Pagrus pagrus* (Tsakogiannis et al., 2018).

**Supplementary Table 3.** The list of DEGs (differentially expressed genes) with known gene name. log_2_FC = log_2_ Fold Change.

| Gene | DESeq2 baseMean | | log_2_FC | adjusted p-value |
| --- | --- | --- | --- | --- |
|  | Control fish  (Maleness) | Testis-removed fish  (Femaleness) | (> 1 or < -1) | (< 0.05) |
| *h3f3c* | 103 | 14745 | 7.16 | 1.70E-63 |
| *paics* | 4 | 283 | 6.08 | 1.24E-03 |
| *slc12a9* | 27 | 1458 | 5.77 | 2.15E-04 |
| *med1* | 11 | 277 | 4.63 | 3.31E-03 |
| *thsd7b* | 4 | 66 | 4.00 | 2.11E-03 |
| *arih2* | 85 | 1189 | 3.80 | 3.51E-02 |
| *cd277* | 60 | 458 | 2.94 | 7.72E-30 |
| *slc25a11* | 28 | 212 | 2.92 | 4.97E-07 |
| *ddx5* | 8 | 55 | 2.85 | 3.96E-05 |
| *auh* | 35 | 203 | 2.52 | 2.58E-02 |
| *acsf3* | 12 | 41 | 1.84 | 2.11E-02 |
| *rpl12* | 662 | 2278 | 1.78 | 1.20E-03 |
| *sowahc* | 21 | 73 | 1.77 | 6.37E-03 |
| *arg1* | 1477 | 4785 | 1.70 | 1.24E-05 |
| *setd4* | 71 | 215 | 1.60 | 6.13E-03 |
| *wdr20* | 27 | 80 | 1.59 | 3.04E-02 |
| *pex12* | 116 | 315 | 1.44 | 1.38E-02 |
| *pdcd2l* | 34 | 90 | 1.41 | 4.94E-02 |
| *dclre1a* | 430 | 1131 | 1.40 | 3.66E-03 |
| *c2orf49* | 51 | 125 | 1.29 | 1.17E-02 |
| *arl9* | 79 | 177 | 1.17 | 4.94E-02 |
| *mid1ip1* | 4325 | 9638 | 1.16 | 4.40E-05 |
| *nus1* | 130 | 287 | 1.14 | 3.57E-02 |
| *hccs* | 294 | 637 | 1.12 | 1.35E-02 |
| *hspbap1* | 94 | 194 | 1.03 | 6.17E-03 |
| *pttg1* | 31878 | 13774 | -1.21 | 9.06E-04 |
| *junb* | 177 | 70 | -1.35 | 5.84E-03 |
| *mr1* | 1392 | 545 | -1.36 | 1.78E-02 |
| *agpat3* | 5874 | 2210 | -1.41 | 1.77E-02 |
| *vkorc1* | 536 | 194 | -1.47 | 3.94E-03 |
| *stag3* | 988 | 344 | -1.52 | 7.36E-03 |
| *rdh14* | 342 | 106 | -1.71 | 3.25E-02 |
| *ptk7* | 122 | 36 | -1.74 | 4.94E-02 |
| *nlrp12* | 55 | 10 | -2.40 | 3.16E-02 |
| *ncam1* | 382 | 66 | -2.53 | 9.94E-03 |
| *rtl1* | 64 | 10 | -2.79 | 2.94E-04 |
| *csrp3* | 83 | 9 | -3.24 | 1.18E-02 |
| *kcnmb4* | 105 | 10 | -3.30 | 3.31E-03 |
| *rps2* | 344 | 31 | -3.46 | 2.09E-03 |
| *lnpep* | 630 | 56 | -3.50 | 4.51E-05 |
| *tap2* | 284 | 25 | -3.51 | 3.62E-02 |
| *loxl3* | 135 | 10 | -3.73 | 8.56E-03 |
| *ccnb2* | 2795 | 167 | -4.07 | 1.46E-02 |
| *c3* | 47 | 2 | -4.38 | 3.85E-02 |
| *rpl9* | 11113 | 501 | -4.47 | 4.64E-03 |
| *cdk16* | 208 | 9 | -4.57 | 3.16E-03 |
| *pros1* | 335 | 14 | -4.63 | 1.44E-04 |
| *zyx* | 969 | 31 | -4.95 | 1.21E-06 |
| *trim35* | 55 | 1 | -5.37 | 6.97E-07 |
| *ptplad2* | 157 | 4 | -5.40 | 3.31E-03 |
| *hnrnpa1* | 576 | 5 | -6.94 | 6.74E-05 |
| *plcd3* | 293 | 2 | -7.23 | 6.66E-24 |
| *epas1* | 391 | 2 | -7.42 | 1.17E-03 |
| *zp3* | 7955 | 19 | -8.74 | 3.65E-60 |
| *lmna* | 104 | 0 | -9.09 | 5.77E-07 |
